# The mc^2^-CMX vaccine induces an enhanced immune response against *Mycobacterium tuberculosis* compared to Bacillus Calmette-Guérin but with similar lung inflammatory effects

**DOI:** 10.1590/0074-02760150411

**Published:** 2016-04

**Authors:** Fábio Muniz de Oliveira, Monalisa Martins Trentini, Ana Paula Junqueira-Kipnis, André Kipnis

**Affiliations:** 1 Universidade Federal de Goiás, Instituto de Patologia Tropical e Saúde Pública, Laboratório de Bacteriologia Molecular, Goiânia, GO, Brasil; 2 Universidade Federal de Goiás, Instituto de Patologia Tropical e Saúde Pública, Laboratório de Imunopatologia das Doenças Infecciosas, Goiânia, GO, Brasil

**Keywords:** recombinant vaccine, tuberculosis, inflammation, mouse

## Abstract

Although the attenuated *Mycobacterium bovis* Bacillus Calmette-Guérin (BCG) vaccine has been used since 1921, tuberculosis (TB) control still proceeds at a slow pace. The main reason is the variable efficacy of BCG protection against TB among adults, which ranges from 0-80%. Subsequently, the mc^2^-CMX vaccine was developed with promising results. Nonetheless, this recombinant vaccine needs to be compared to the standard BCG vaccine. The objective of this study was to evaluate the immune response induced by mc^2^-CMX and compare it to the response generated by BCG. BALB/c mice were immunised with both vaccines and challenged with*Mycobacterium tuberculosis* (Mtb). The immune and inflammatory responses were evaluated by ELISA, flow cytometry, and histopathology. Mice vaccinated with mc^2^-CMX and challenged with Mtb induced an increase in the IgG1 and IgG2 levels against CMX as well as recalled specific CD4^+^ T-cells that produced T-helper 1 cytokines in the lungs and spleen compared with BCG vaccinated and challenged mice. Both vaccines reduced the lung inflammatory pathology induced by the Mtb infection. The mc^2^-CMX vaccine induces a humoral and cellular response that is superior to BCG and is efficiently recalled after challenge with Mtb, although both vaccines induced similar inflammatory reductions.

Tuberculosis (TB) has been studied since the 460 years B.C. (Benedek 2004); however, during the current post-genomic era, TB remains one of the most important public health problems worldwide. Nine million new TB cases were reported in 2013 and, despite the significant advances in treating the disease over the last few decades, 1.5 million deaths due to *Mycobacterium tuberculosis* (Mtb), the causative agent of TB, occurred (WHO 2014). Additionally, the global scenario was aggravated by the increasingly high numbers of reported multidrug-resistant (MDR)-TB strains. Of all of the registered TB cases in 2013, 3.5% were due to MDR-TB, representing 480,000 cases that resulted in the deaths of 210,000 individuals (WHO 2014). Therefore, several research groups are seeking alternatives to fight TB, including developing new vaccines, because the best way to overcome a disease is to prevent infection and/or disease development.

Bacillus Calmette-Guérin (BCG) vaccine is very efficient in protecting children against severe forms of TB; however, its efficacy wanes with time and is highly variable among adult individuals (0-80%), the age group with the highest incidence of the disease (Fine 1995, Andersen & Doherty 2005, Kaufmann et al. 2010). In addition to its variable activity, BCG is not recommended for use in human immunodeficiency virus-positive children or those that have a genetic deficiency in interleukin (IL)-12 or interferon (IFN)-g (Ottenhoff et al. 2002, Hesseling et al. 2007). Consequently, it is imperative to control TB by developing vaccines that can replace BCG or boost its protection among those individuals who cannot be vaccinated with it (Ottenhoff & Kaufmann 2012).

The sequencing of Mtb genome promoted the discovery and characterisation of several important mycobacterial proteins that are produced during the infection of the host, which supported and strengthened TB vaccine studies (Cole et al. 1998, Rachman & Kaufmann 2007, Zvi et al. 2008). Additionally, genomic studies identified deleted genes from *Mycobacterium bovis*responsible for the BCG attenuation process, the most important being those within the region of difference 1 (RD1) (Mahairas et al. 1996, Philipp et al. 1996). Consequently, several TB vaccine development approaches have relied in the reintroduction of some of the deleted genes (ESAT-6 and CFP-10 for example) (Kalra et al. 2007, Zhang et al. 2010, Shaban et al. 2013, Bottai et al. 2015). Despite the many studies using those RD1 proteins, several studies have shown that proteins present in both *M. bovis* and Mtb were promising when used as subunit and/or vector vaccines (Wang et al. 2012, Marongiu et al. 2013, Darrah et al. 2014, Trentini et al. 2014, Yuan et al. 2015). We developed a fusion recombinant protein, CMX, composed of the immunodominant epitopes of the Mtb proteins Ag85C, MPT-51, and HspX that was shown to induce a specific immune response in mice (deSousa et al. 2012). These studies indicated the beneficial use of the recombinant fusion CMX protein in the context of a new TB vaccine. In this regard and considering the limitations of BCG, a recombinant vaccine composed of the avirulent strain of *Mycobacterium smegmatis*mc^2^ 155 expressing the recombinant fusion protein CMX (mc^2^-CMX) was constructed. When tested in a murine model, this vaccine induced a specific immune response with protective efficacy against Mtb infection (Junqueira-Kipnis et al. 2013). However, the differences in the immune responses induced by the mc^2^-CMX and BCG vaccines have not yet been studied. The aim of this study was to compare the immune responses induced by the mc^2^-CMX and BCG vaccines.

## MATERIALS AND METHODS

*Animals* - The study was conducted in six-eight-week-old BALB/c mice from the Institute of Tropical Pathology and Public Health at the Federal University of Goiás (UFG), city of Goiânia, state of Goiás, Brazil, animal facilities that were housed in HEPA-filtered racks and fed with water and a standard diet *ad libitum*. The temperature was maintained between 20-24ºC, with a relative humidity between 40-70%, and light/dark cycles of 12 h. The mice were maintained and handled in accordance with the rules of the Brazilian Society of Science in Laboratory Animals. This study was approved by the Ethical Committee of UFG under protocol 229/11.

*Vaccine preparation* - Aliquots of the mc^2^-CMX-vaccine, which were previously produced as described by Junqueira-Kipnis et al. (2013), were removed from the -80ºC freezer and the concentration was adjusted to 1 x 10^8^ colony-forming unit (CFU)/mL with phosphate-buffered saline (PBS) containing 0.05% Tween 80. The same procedure was used to prepare the vaccine inoculum of BCG Moreau; however, the concentration was adjusted to 10^7^ CFU/mL. The control groups received PBS with 0.05% Tween 80. The vaccine diluted for each experiment was plated onto 7H11 media to confirm the inoculum concentration.

*Immunisation* - Sixteen BALB/c mice were divided into four groups of four mice each: PBS, PBS + Mtb (infection), BCG + Mtb, and mc^2^-CMX + Mtb. The PBS, infection, and mc^2^-CMX + Mtb groups received two immunisations (100 mL/immunisation, subcutaneous injections) with an interval of 15 days between injections. The BCG + Mtb group (100 mL/immunisation, subcutaneous injection) received a single immunisation. In all vaccine immunisations, the vaccine inoculum was plated onto 7H11 agar to confirm the concentration.

*Intravenous infection with Mtb H37Rv* - Thirty days after the last immunisation, the animals were challenged with Mtb H37Rv prepared as described byJunqueira-Kipnis et al. (2013). On the day of infection, the inoculum was diluted to a concentration of 10^8^CFU/mL in PBS with 0.05% Tween 80, and 100 mL (10^7^ CFU) was administered intravenously (*via* the retroorbital plexus). Seventy days after infection, the mice were sacrificed to analyse their cellular immune responses and the pathological changes in their lungs.

*ELISA* - Blood samples were collected from the mice in each group 15 days before and 30 days after challenge. The collected blood was incubated for 1 h at 37ºC, centrifuged at 1,200 *g* at 4ºC for 15 min to separate the serum and subsequently stored at -20ºC. To determine the levels of the anti-CMX antibodies of IgG1 and IgG2a classes in the serum, an ELISA was performed and optimised as described by Junqueira-Kipnis et al. (2013).

*Lung and spleen cell preparation* - Seventy days after infection, all mice were euthanised by cervical dislocation and their lungs and spleens were collected. The lung digestion was performed in a solution of type IV DNase (30 µg/mL) (Sigma-Aldrich, USA) and Collagenase III (0.7 mg/mL) (Sigma-Aldrich) for 30 min at 37ºC. The lung cell suspension was obtained by passing the digested tissue through a 70 µm cell strainer. The erythrocytes were lysed with lysis solution (0.15 M NH_4_Cl and 10 mM KHCO_3_) and the cells were then washed and resuspended in complete RPMI (cRPMI) medium (Junqueira-Kipnis et al. 2003). Finally, the viable cells were counted and adjusted to a density of 1 x 10^6^ cells/mL. The splenocytes were obtained after passing the organ through a 70 µm cell strainer (BD Biosciences, USA) and immediately resuspended in RPMI medium (RPMI-1640) (GIBCO, USA). The erythrocytes were lysed with lysis solution and the cells were then washed and resuspended in cRPMI medium. Finally, the viable cells were counted and adjusted to a density of 1 x 10^6^ cells/mL.

*Intracellular cytokine profile in the lung and spleen* - To identify the cytokines that were produced by the CD4^+^ T-cells in the lung and spleen, the cells were cultured without stimulation (cRPMI medium) during 4 h at 37ºC in a 5% CO_2_ incubator. Next, 3 µM monensin was added (BD Biosciences) for an additional 6 h. Then, the cells were stained with an anti-CD4-PerCP antibody (BD Pharmingen^®^, USA) and fixed and permeabilised with Perm Fix/Perm Wash (BD Pharmingen^®^). The cells were then stained with the following antibodies for 30 min: IL-2-PE, TNF-α-FITC, and IFN-γ-APC or IgG2a/IgG1 isotypes control (all antibodies used were from eBioscience^®^, USA). All analyses were performed on 50,000 events acquired in a BD Biosciences FACSCalibur flow cytometer (Araújo Jorge Hospital, Goiânia) and the data were analysed with the FlowJo 8.7 software. The lymphocytes were selected based on their size (forward scatter) and granularity (side scatter).

*Histopathological analysis* - For the histopathological analysis of the lungs, the right caudal lobes of the lungs from each mouse were collected 70 days after infection and fixed with 10% buffered formalin. The following parameters were evaluated under the microscope at 5X, 10X, 20X, 40X, and 100X magnifications: the intensity of the inflammatory infiltrate and the presence or absence of foamy macrophages and necrotic areas.

A score of zero was attributed to histological sections that did not present any lesions or inflammatory foci and the lung architecture was preserved. A value from 1-4 was attributed to samples that presented a few inflammatory foci and foamy mononuclear macrophages, with 1 being the minimal number of events in the fields and 4 when one or two events were observed in several fields. The presence of lesions, inflammatory foci, diffuse mononuclear infiltrates, and foamy macrophages were given a score between 5-7, where a score of 5 was received when three-five events per field was observed and a score of 7 represented samples with six-eight events per field. Histological samples that presented lesions, inflammatory foci, a moderate, diffuse mononuclear infiltrate, foamy macrophages, and necrosis received scores from 8-10 with a score of 8 attributed to samples presenting eight-10 inflammatory foci per field with little necrosis and a score of 10 attributed to samples that exhibited accumulated lesions and necrosis associated with the foci and the loss of the lung architecture.

*Statistical analysis* - The results were tabulated with Excel (v.14.3.4, 2011 for Mac) and the Prism software (v.6.0a, GraphPad). The differences between groups were assessed with a two-tailed Student’s *t* test after a nonparametric (Mann-Whitney *U*) test. The results were considered significantly different when p < 0.05.

## RESULTS

**Fig. 1:**
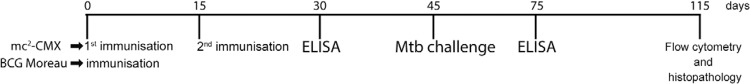
: timeline scheme of the experimental design. Mice were immunised once with Bacillus Calmette-Guérin (BCG) [106 colony-forming unit (CFU)/mouse] or twice with mc2-CMX (107 CFU/mouse) with 15 days interval. Thirty days after first immunisation, blood was collected from mice and assayed in an ELISA for IgG1 and IgG2a antibodies against CMX. Forty-five days after initial immunisation, mice were intravenously challenged with *Mycobacterium tuberculosis* (Mtb) H37Rv strain (107 CFU/mouse). Thirty days after challenge, blood was collected for ELISA. Seventy days after challenge, mice were euthanised and organs were removed for flow cytometry and histopathological analyses.

**Fig. 2:**
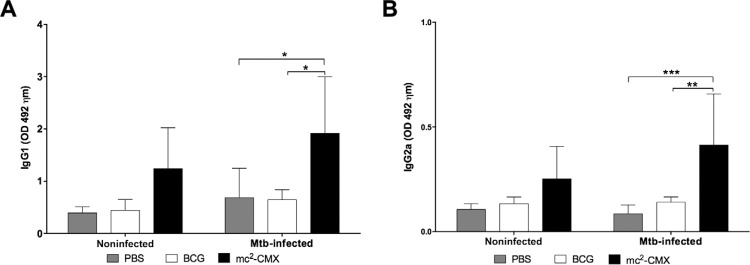
: a specific humoral immune response is induced in BALB/c mice by the Bacillus Calmette-Guérin (BCG) and mc2-CMX vaccines prior to and after the *Mycobacterium tuberculosis* (Mtb) challenge. A: serum levels of the CMX-specific IgG1 class antibodies before (noninfected) and after Mtb challenge (Mtb-infected); B: serum levels of the CMX-specific IgG2a class antibodies before (noninfected) and after Mtb challenge (Mtb-infected); OD: optical density; PBS: phosphate-buffered saline; *: p < 0.05; **: p < 0.01; ***: p < 0.001: significant differences between groups.

*The humoral immune response against CMX induced by the mc* ^*2*^ *-CMX vaccine is two times higher than that induced by BCG* - Because the proteins chosen to comprise the recombinant vaccine are produced by most of the mycobacteria species, it is necessary to know if there is a difference in the immunogenicity of recombinant fused protein in two different live vectors: *M. smegmatis* and *M. bovis* BCG. Fig. 1 depicts the timeline of experimental procedures as well as vaccination and Mtb challenge (Fig. 1). Fifteen days after the last immunisation, blood samples were collected from all mice to perform an ELISA. As shown in Fig. 2A,B, mice immunised with the mc^2^-CMX vaccine had similar serum levels of the anti-CMX antibodies of both the IgG1 and IgG2a classes compared to the group immunised with BCG or PBS (Fig. 2).

To assess whether Mtb infection could recall the immune response induced by previous vaccination, blood samples were collected from all animals at 30 days after the infection and the levels of the anti-CMX antibodies were determined. As shown inFig. 2A, B, the levels of the CMX-specific antibodies of both the IgG1 and IgG2a classes were significantly higher in the mice immunised with the mc^2^-CMX vaccine compared to the animals from the PBS or BCG groups (Fig. 2). These results show that the mc^2^-CMX-vaccine induced a specific humoral immune response against CMX only after challenge, while this was not observed after vaccination with BCG.

**Fig. 3:**
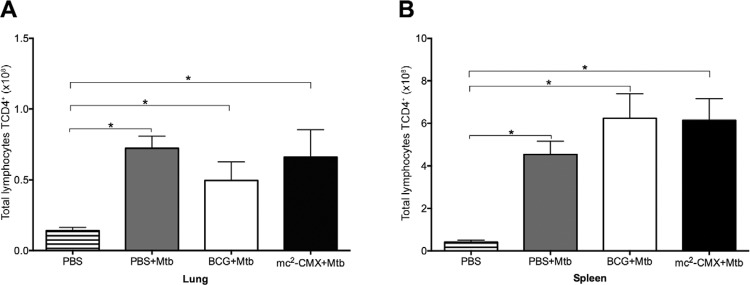
: CD4+ T lymphocytes in the lungs and spleen of the mice. The mice were immunised and challenged with *Mycobacterium tuberculosis* (Mtb) 30 days after the last immunisation. Seventy days after challenge, the lungs and spleen were collected and analysed by flow cytometry. The cells were cultivated ex vivo without stimuli for 4 h at 37ºC. The total number of CD4+ T lymphocytes in the lungs (A) and spleen (B) were determined. Mice that were not immunised or infected were used as controls [phosphate-buffered saline (PBS)]. BCG: Bacillus Calmette-Guérin; *: p < 0.05 indicates a significant difference between groups.

*The mc* ^*2*^ *-CMX-vaccine induces a greater number of CD4* ^*+*^ *T-cells that produce IFN-*g *and TNF-*a *in the lungs of the infected mice compared to BCG* - The control of an Mtb infection is mainly related to the phenotypic profile of the cells that migrate to the site of infection and their effector activity (Cooper 2009). Therefore, we assessed if prior immunisation with mc^2^-CMX or BCG could modulate the frequency of these cells in the lung and spleen 70 days after Mtb infection. As shown in Fig. 3A, Mtb infection alone was able to induce an increased migration of CD4^+^ T lymphocytes to the lungs of the mice, regardless of their immunisation status (Fig. 3A). The same result was observed in the spleen (Fig. 3B), where there was an increase in these populations in all groups challenged with Mtb compared to the nonchallenged group.

**Fig. 4:**
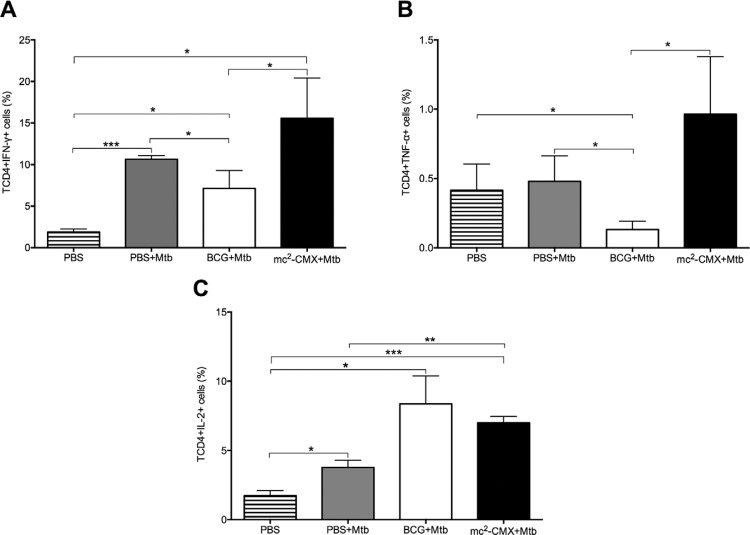
: percentage of CD4+ T lymphocytes that produce inflammatory cytokines in the lungs of the *Mycobacterium tuberculosis* (Mtb)-challenged mice. The mice were immunised and challenged with Mtb 30 days after the last immunisation. Seventy days after challenge, the lungs were collected and analysed by flow cytometry. The cells were cultivated ex vivo without stimuli for 4 h at 37ºC. The number of interferon (IFN)-γ-positive (A), tumour necrosis factor (TNF)-α-positive (B), and interleukin (IL)-2-positive CD4+ T lymphocytes (C) was determined. Comparisons between groups were performed by *t* test*.* BCG: Bacillus Calmette-Guérin; PBS: phosphate-buffered saline; *: p < 0.05 indicates a significant difference between groups.

Cytokine production by CD4^+^ effector T lymphocytes is an important factor in the control of Mtb infection, particularly when they exhibit characteristics of a T-helper (Th)1-type response, such as IFN-g, TNF-a, and IL-2 production. Thus, we evaluated the profile of the cytokines produced by the CD4^+^ T-cells in the lungs of the mice immunised with each vaccine and challenged with Mtb (gating strategy of representative dot plots is shown in Supplementary Figure). As shown inFig. 4A, the mice immunised with mc^2^-CMX vaccine had a significantly higher percentage of IFN-g producing cells compared to the group immunised with BCG; however, there was no difference compared to the infected group (PBS + Mtb) (Fig. 4A). The percentage of cells producing TNF-a was also significantly higher in the group immunised with the mc^2^-CMX vaccine compared to the BCG group (Fig. 4B), but this increase was not significantly different compared to the group that received PBS (PBS + Mtb). In determining the percentages of CD4^+^ T-cells that produced IL-2, the mice immunised with mc^2^-CMX and BCG had significantly higher levels than the PBS group, but only the mc^2^-CMX had significantly higher levels when compared to the infection group (Fig. 4C).

**Fig. 5:**
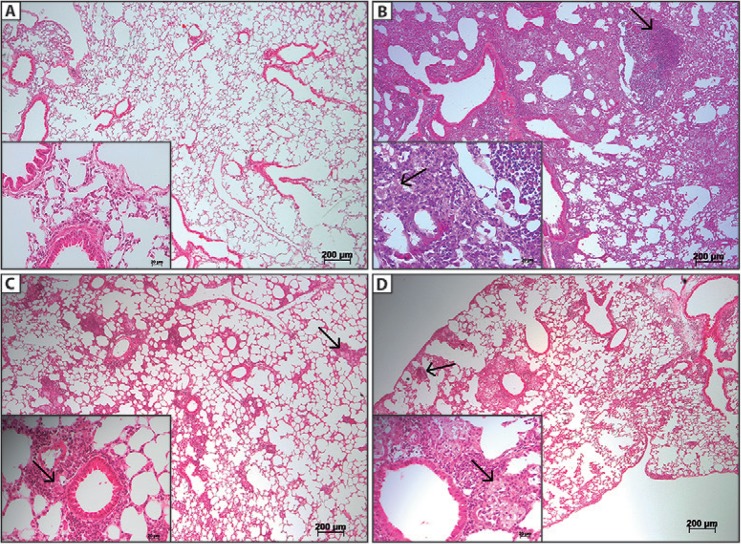
: immunisation with the mc2-CMX vaccine prevented the lung lesions caused by *Mycobacterium tuberculosis* (Mtb) infection, similar to the Bacillus Calmette-Guérin (BCG) vaccine. Histopathological analysis of the lungs from the BALB/c mice 70 days after the H37Rv Mtb infection. A: phosphate-buffered saline (PBS) (noninfected group); B: nonimmunised and infected group (PBS + Mtb). The arrows indicate the mononuclear infiltrate (5X magnification) and foamy macrophages (40X magnification); C: mouse immunised with the BCG vaccine and challenged with Mtb. The arrows indicate the inflammatory infiltrates (5X magnification) and necrotic cells (40X magnification); D: mouse immunised with mc2-CMX challenged with Mtb. The arrows indicate the mononuclear infiltrate (up to 5X) and foamy macrophages. The slides were stained with haematoxylin and eosin and analysed by light microscopy (Axio scope.A1; Carl Zeiss) using AxioVision software v.4.7.

*Both mc* ^*2*^ *-CMX and BCG vaccines reduce the severity of the lung lesions in BALB/c mice infected with Mtb* - After 70 days of infection, the lungs were collected from all mice for histological evaluations. In the infection group (PBS + Mtb) (Fig. 5B), the intravenous challenge with Mtb induced severe and diffuse lung inflammation, which led to the consolidation of the lung parenchyma. Large inflammatory agglomerates containing neutrophils, mononuclear cells, and foamy macrophages were also present (Fig. 5B). In contrast, the mice immunised with the BCG or mc^2^-CMX vaccines had preserved the lung parenchyma architecture, with few inflammatory foci compared to the infection group (Fig. 5C,D).

**Fig. 6:**
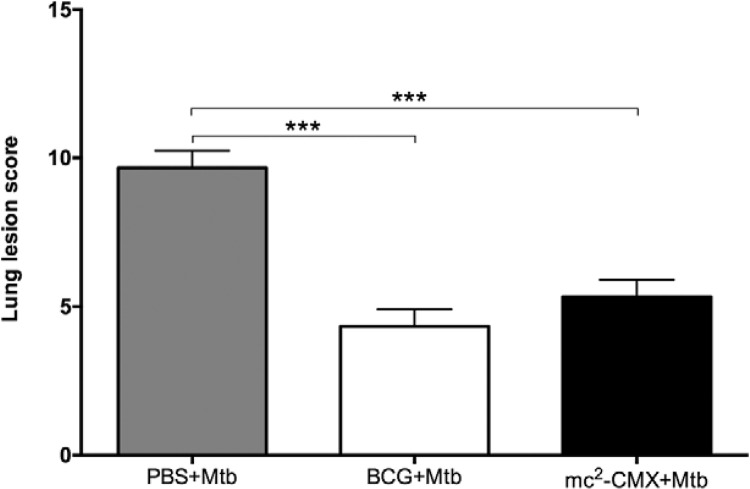
: lung lesion scores in the animal groups that were challenged with*Mycobacterium tuberculosis* (Mtb) infection. The mice were immunised with the Bacillus Calmette-Guérin (BCG) or mc2-CMX vaccines (BCG + Mtb or mc2 - CMX + Mtb, respectively). Nonimmunised mice were also infected with Mtb [phosphate-buffered saline (PBS) + Mtb]. Seventy days after infection, the lungs from the mice were collected and analysed by haematoxylin and eosin staining. The lesion scores were determined based on the number of inflammatory foci, lesions, foamy macrophages, inflammatory cells, and the presence of necrosis. The analysis was performed by optical microscopy (Axio scope.A1; Carl Zeiss) using AxioVision software v.4.7. ***: p < 0.001 indicates a significant difference between groups.

To enhance the comparisons of the immune response induced by the vaccines following Mtb infection, a score was attributed to the lung lesions observed in each group. Significantly more lesions were observed in the infection group (PBS + Mtb) compared to the groups immunised with the BCG or mc^2^-CMX vaccines (Fig. 6), but there were no differences in the lesion scores of the two vaccinated groups. The responses induced by the BCG and mc^2^-CMX vaccines were able to preserve the lung architecture, significantly avoiding the immunopathology of Mtb infection.

## DISCUSSION

In this study, we compared the immune response and efficacy of the recombinant vaccine with the widely used BCG Moreau vaccine. The mc^2^ *-*CMX vaccine increased the production of antibodies specific for the CMX protein in BALB/c mice compared to the BCG vaccine. When assessing the immunological profile induced by the recombinant vaccine against the challenge with Mtb, we observed significant differences in the IFN-g and TNF-a levels in the mc^2^ *-*CMX-treated mice compared to the BCG-treated mice. Although the vaccines induced different immunological profiles, they were both effective in reducing lung injury in BALB/c mice.

Although they are crucial in the control of infections, such as *Leishmania major* infections (Woelbing et al. 2006), the role of antibodies in the immune response against TB is not clear. However, studies have shown the importance of B-cells in the generation of a protective immune response against Mtb infection (Maglione et al. 2007, Maglione & Chan 2009). Torrado et al. (2013)observed that mice deficient in antibody maturation are more susceptible to Mtb infection, but they could not determine the role of the antibodies. However, increased production of IL-10 was observed in the deficient mice, resulting in an increased susceptibility to infection. This shows that the induction of a humoral immune response is important in developing a protective immune response. However, by itself, it may not be effective in infection control, although it is involved in the modulation of the immune response by aiding in T-cell proliferation, differentiation, and survival (Abebe & Bjune 2009, Torrado et al. 2013).

In our studies, the mc^2^-CMX-vaccine was able to induce significant levels of CMX-specific antibodies of the IgG1 and IgG2a classes (Fig. 2) compared to the BCG vaccine. Interestingly, BCG contains the protein antigens present in CMX (Ag85C, MPT-51, and HspX); however, it did not induce the production of anti-CMX antibodies. The lack of the humoral immune response in mice immunised with the BCG vaccine may have been due to the reduced expression of the HspX protein in this vaccine, as several studies have shown that BCG does not induce a specific immune response against HspX in humans or mice (Geluk et al. 2007, Shi et al. 2010, Spratt et al. 2010). Furthermore, de Sousa et al. (2012) demonstrated that the major rCMX-induced humoral immune response in mice was towards the HspX protein.

IFN-g and TNF-a present key roles in TB protection, and the Th1 subpopulation of CD4^+^ T-cells secrete those cytokines that activate macrophages (Cooper 2009, Bold & Ernst 2012) and contribute to the migration of these cells to the site of infection, particularly the lungs. IFN-g and TNF-a can induce the microbicidal actions of macrophages, such as the production of reactive oxygen and nitrogen intermediates and autophagy induction (Flynn et al. 1993, Saunders et al. 2002, Gutierrez et al. 2004). Another protective mechanism of TNF-a is to aid in the development of granulomas (Kindler et al. 1989, Flynn et al. 1995, Ramakrishnan 2012).

In this study, we observed a migration of CD4^+^ T lymphocytes to the lungs in all groups challenged with Mtb, demonstrating that infection alone was capable of inducing the migration of these cells (Fig. 3). However, when evaluating the profile of the Th1 cytokines produced by these cells in the lung, we found that IFN-g and TNF-a were increased in the mice immunised with the mc^2^-CMX vaccine compared to the group immunised with the BCG vaccine (Fig. 3A, B). Similar results were obtained by Zhang et al. (2010), who demonstrated that IFN-g production by the CD4^+^ T-cells was increased in the group of mice that were immunised with *M. smegmatis* expressing a CFP10-ESAT6 fusion protein compared to the BCG group (Zhang et al. 2010).

Although IFN-g is crucial for Mtb infection protection, some studies have shown that the BCG-induced protection is not only IFN-g-dependent, because BCG-vaccinated IFN-g-deficient mice challenged with Mtb exhibited better infection control than the mice depleted of CD4^+^ T lymphocytes (Cowley & Elkins 2003, Elias et al. 2005, Abebe 2012). Therefore, CD4^+^ T-cells can control the Mtb infection through mechanisms that are not exclusively dependent on IFN-g; alternatively, other cells, such as natural killer (NK) cells, can control the infection (Cowley & Elkins 2003, Mittrucker et al. 2007, Abebe 2012). We observed this phenomenon in our studies, because of the mice immunised with the BCG vaccine showed a significant reduction in lung injury, similar to mice immunised with mc^2^-CMX (Figs 5, 6). Additionally, mice vaccinated with mc^2^-CMX and challenged with Mtb presented higher levels of IL-2 than infected animals; therefore IL-2 could be involved in those protective mechanisms. IL-2 induces the activation and proliferation of Th1 CD4^+^ T-cells and CD8^+^ T-cells and consequently results in the suppression of Mtb replication (Orme 1993, Kim et al. 2000, Williams et al. 2006, Seder et al. 2008). Furthermore, IL-2 can activate NK cells to produce IFN-g and consequently increase Mtb elimination by macrophages (Esin et al. 2013). It seems that BCG vaccinated mice and challenged with Mtb also show the same trend in increase of IL-2, however future work should be done to confirm this hypothesis using a higher number of animals (Fig. 4C).

Polyfunctional cells has been associated with the protection induced by vaccines (Darrah et al. 2007, Lindenstrom et al. 2009, Derrick et al. 2011), therefore BCG protection may comprise the development of Th cells that express more than one cytokine. Our group showed that Mtb infection significantly reduces the frequency of triple positive CD4^+^T-cells in the spleen of nonimmunised mice that was not observed in mice previously vaccinated with mc^2^-CMX, suggesting the importance of those cells in TB protection (Junqueira-Kipnis et al. 2013). This hypothesis is corroborated by the algorithm developed by Boyd et al. (2015). Here we hypothesise that polyfunctional T-cells could compensate for the lower levels of IFN-γ positive cells induced by BCG vaccination.

The lungs of the mice from the infection group (PBS + Mtb) showed significantly increased percentages of Th1 cytokines (Fig. 4C) accompanied by excessive tissue injury with inflammatory lymphocytic and macrophagic clusters, characteristics of the lack of infection control (Figs 5, 6, and data not shown). This phenomenon is likely related to the fact that a protective immune response to TB is not limited to or solely dependent on the production of Th1 cytokines, but on the balance of the immune response as a whole (Walzl et al. 2011). Previous studies using the wild type strain of the *M. smegmatis* vaccine showed that, even though it was capable of inducing similar numbers of CD4^+^ T-cells that produce Th1-type cytokines, as observed in this study using the mice immunised with the mc^2^-CMX vaccine, the former was not able to reduce the bacterial load in the lungs of the BALB/c mice. This could be primarily due to the capacity of the mc^2^-CMX vaccine in inducing IL-17 production from the CD4^+^T-cells in the lungs of the BALB/c mice, a phenomenon that was also observed for the*M. smegmatis* Immune Killing Evasion - CMX vaccine (Junqueira-Kipnis et al. 2013).

The recombinant CMX fusion protein has been shown to play an effective role in improving vaccine efficiency. Studies by da Costa et al. (2014) showed that the addition of this protein to the BCG vaccine increased its ability to induce the production of both IL-17 and Th1-type cytokines (IFN-g and TNF-a) by the CD4^+^ T-cells (da Costa et al. 2014). Thus, we believe that the addition of the CMX protein induces the development of a balanced immune response that is capable of improving the control of Mtb infection. Thus, the immune response induced by a vaccine that aims to replace or improve BCG should not simply increase the immune response, but instead provide a more balanced immune response by optimising the host defense mechanisms and reducing the inflammatory lesions, thus improving infection control and preserving the architecture of the infected organ (Ottenhoff 2012).

This study demonstrated that both mc^2^-CMX and BCG vaccines were able to prevent the deleterious effects in the lungs of mice infected with Mtb, likely through different immune mechanisms than BCG. Moreover, this path should be followed to obtain a vaccine that can replace or enhance BCG by providing immunogenic properties that are absent in the BCG vaccine, such as the induction of a humoral immune response.
